# Immunomorphologic Manifestations in Mice Liver Infected with Influenza A/H5N1, A/Goose/Krasnoozerskoye/627/05 Strain

**DOI:** 10.1155/2013/342686

**Published:** 2013-12-23

**Authors:** Oxana V. Potapova, Tatyana V. Sharkova, Vyacheslav A. Shkurupiy, Alexander M. Shestopalov

**Affiliations:** FSBI Research Center of Clinical and Experimental Medicine, SB RAMS, Timakova Street 2, Novosibirsk 630117, Russia

## Abstract

Highly pathogenic avian influenza H5N1 (HPAI H5N1) viruses can infect mammals, including humans, causing severe systemic disease with the inhibition of the immune system and a high mortality rate. In conditions of lymphoid tissue depletion, the liver plays an important role in host defence against viruses. The changes in mice liver infected with HPAI H5N1 virus A/goose/Krasnoozerskoye/627/05 have been studied. It has been shown that the virus persistence in the liver leads to the expression of proinflammatory cytokines (TNF-**α**, IL-6) and intracellular proteases (lysozyme, cathepsin D, and myeloperoxidase) by Kupffer cells. Defective antiviral response exacerbates destructive processes in the liver accelerating the development of liver failure.

## 1. Introduction

Influenza takes a special place in the human infectious pathology because it has no equivalent in prevalence and incidence of diseases. The causative agent of the highly pathogenic avian influenza A/H5N1 (HPAI H5N1) is of particular interest. Its characteristic features are wide geographical distribution and high pathogenicity to humans causing the severe disease with high mortality in the absence of specific immunity in the human population [1–3]. There is a constant threat of a pandemic, due to the constant evolution of influenza viruses, the high population density in areas of active circulation of HPAI H5N1, its ability to direct transmission from birds to humans, and the possibility of reassortment with viruses adapted in the human population [4–6].

An infectious disease caused by HPAI H5N1 viruses occurs with damage to many organs and systems of the organism and severe symptoms of intoxication [7, 8]. When studying the distribution of HPAI H5N1 viruses in tissues of internal organs of infected animals in a number of studies, it was shown that they are able to replicate not only in the lung, but also in extrapulmonary organs including liver, kidney, spleen, and brain [9–11]. The incubation period typical for HPAI H5N1 is too brief for the occurrence of primary humoral response [12], and the initial period of the disease occurs with the inhibition of the interferon response and reduction of interferon in the blood [10]. At the same time, many researchers have reported significant depletion of lymphoid tissue as a result of massive apoptosis of lymphocytes under the infection [13, 14]. In this connection, a special role acquires nonspecific protection mechanisms of the organism, particularly cells of the mononuclear phagocyte system, a significant pool of which is concentrated in the lungs and liver [12]. Macrophages can phagocytize viruses, destroying them [12, 15]; however, in cases of infection with highly pathogenic viruses, including HPAI H5N1, their successful replication occurs in macrophages with the development of cytopathic effect expressed in many organs, which often results in fatal outcome [16].

Because of pneumotropic influenza viruses, most studies are devoted to the investigation of morphological manifestations of HPAI H5N1 in the lungs [1, 2, 8, 16, 17]. However, the liver being the central organ of detoxification and immunity is an important barrier to the spread of viruses in the organism. Kupffer cells, which are the resident macrophages of the liver, in addition to phagocytic function, have a high secretory activity. Among the products of secretion the most important are inflammatory (IL-1, IL-6, TNF-*α*, IL-8, IL-12) and anti-inflammatory (IL-10, TGF) cytokines [18].

Due to the developed lysosomal apparatus, macrophages are showing microbicidal activity by the secretion of lysosomal proteases. In the conditions of viral infections the most important are proteolytic enzymes from the group of endopeptidase (lysozyme, cathepsin D, and myeloperoxidase), asking for intracellular disintegration of the protein structures of viruses [18, 19].

Thus, the high pathogenicity of HPAI H5N1 in mammals is associated with the features of immune response. Structural and functional state of the liver cells play the important role in this process. Aforesaid the purpose of this research was to study the structural and functional changes in the cells of the liver (Kupffer cells, hepatocytes, and endothelial cells) of mice infected with HPAI H5N1 virus A/goose/Krasnoozerskoye/627/05.

## 2. Materials and Methods

The study has been conducted on 240 6–8-week-old outbred white male mice from the laboratory animal nursery of FBRI SRC VB Vector. Animals were housed in standard conditions with free access to food and water. Period of animal adaptation to housing conditions before the experiment was two weeks. The investigation was conducted in accordance with the Declaration of Helsinki international principles. The mice were taken out of the experiment through the dislocation of the cervical spine vertebrae under ether narcosis.

A/goose/Krasnoozerskoye/627/05 (H5N1) influenza virus (hereinafter referred to as A/H5N1 virus), isolated from the lung of a goose which died in September 2005 during avian influenza epizootic in Krasnoozerskoe village, Novosibirsk Region, Russia, was used as infectious agent. Mice were intranasally infected with 10 MLD_50_ of A/H5N1.

Experimental works with studied HPAI H5N1 virus strain have been performed in accordance with sanitary regulations on safe work with microorganisms of III-IV pathogenicity group and helminthes. The study has been conducted in three experiments on 80 mice in each—first group (control) consisted of intact animals (20 mice) and the second group consisted of 60 mice infected with A/goose/Krasnoozerskoye/627/05 (H5N1) influenza virus.

Samples of liver of animals infected with A/H5N1 were obtained 1, 3, 6, and 10 days after infection, 10 animals in each experimental period. Control animals were withdrawn from the experiment on the 1st and 10th day. Specimens were fixed in 10% formalin, dehydrated in alcohols of increasing concentrations, and embedded in paraffin pouring mixture “HISTAMIX” (“BioVitrum,” Russia). Sections of 3 micron thickness were prepared on a rotary microtome HM355S (“Microm,” Germany) and stained with hematoxylin and eosin by standard procedure and by immunohistochemical (IHC) method.

Immunohistochemical (IHC) analysis was performed by using indirect streptavidin-peroxidase method with specific primary antibodies against influenza A antigen (Inf A/FITC (“Abcam”)), TNF-*α* (“DBS”), and IL-6 (“Novocastra”) and lysosomal enzymes (Cathepsin D (“DBS”), Myeloperoxidase (“DBS”), and Lysozyme (“DBS”)). For IHC studies sections were dewaxed and rehydrated. After antigen unmasking in a microwave oven at 700 W power for 20–25 minutes and washing with distilled water, phosphate buffer, endogenous peroxidase was blocked within 5 minutes. Exposure time to the primary antibodies was 30–45 minutes at 37°C. Sections were incubated with streptavidin-peroxidase complex, DAB-substrate and further counterstained with Mayer's hematoxylin. To visualize the antibodies “NovoLink” detection system (“Novocastra”) was used.

Morphometric study of tissue structural elements was conducted using closed test system consisting of 100 points, square 3.64 × 10^5^ *μ*m^2^. There were registered volume density (*V*_*v*_) of destructive changes (as the sum of dystrophy and necrosis) and inflammatory infiltrates in the liver and the numerical density (*N*_ai_) of cells expressing studied IHC markers [20]. Statistical analysis of the results was performed using the statistical analysis package Microsoft Office Excel 2007 and standard software package STATISTICA v.6. The arithmetic mean value (*M*) and standard error of the mean (*m*) were determined. To identify the probability of significance of differences of compared average values we used Student's *t*-test. Differences were considered statistically significant at the 5% significance level (*P* < 0.05).

## 3. Results and Discussion

In experimental infection of mice with influenza virus A/H5N1 A/goose/Krasnoozerskoye/627/05 we observed a high mortality rate of 75%. The death of animals was registered in 7–11 days after infection. The maximum number of dead animals was recorded on day 8 of the experiment.

In previous virological studies, it has been shown that the selected isolate of HPAI H5N1 A/goose/Krasnoozerskoye/627/05 is highly pathogenic for mice and is capable of replicating in many organs, including the liver, without prior adaptation [10].

**Figure 1:**
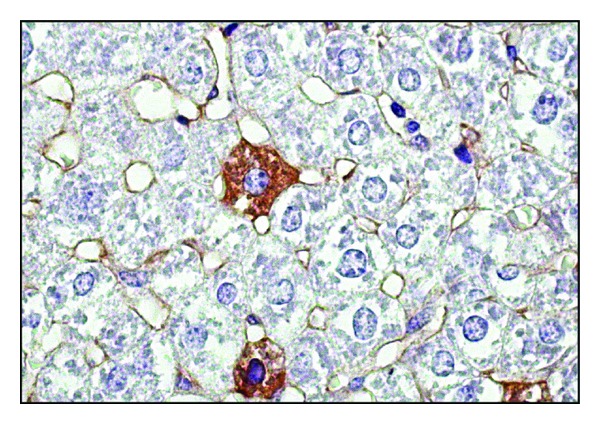
Influenza A virus antigen expression by Kupffer cells, endothelial cells, and hepatocytes of the liver of mice infected with A/goose/Krasnoozerskoye/627/05 (A/H5N1) influenza virus. One day after infection. Immunohistochemical analysis. Magnitude ×1000.

**Figure 2:**
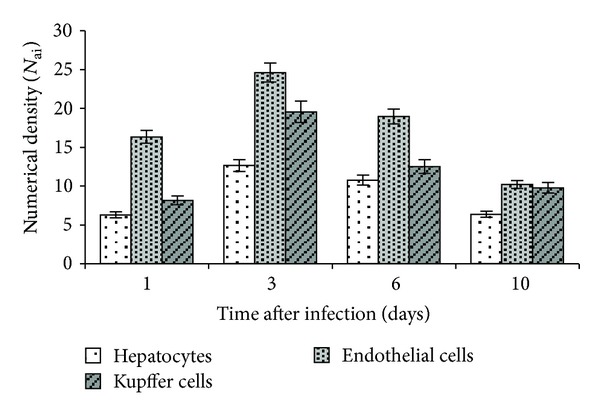
Numerical density of cells expressing influenza A virus antigen in the liver of mice infected with influenza virus A/goose/Krasnoozerskoye/627/05 (A/H5N1).

Visualization of viral antigen by IHC in the liver of infected mice showed that on day 1 of the experiment, and thereafter, the highest rate of replication of the A/H5N1 influenza virus was reported in sinusoidal endothelial cells and Kupffer cells (Figures 1 and 2), which was associated with their phagocytosis function. The maximum number of cells with signs of A/H5N1 influenza virus replication in mice liver was detected on day 3 of infection. The preferential detection of viral antigen in the sinusoidal cells is probably an evidence of the increasing viremia due to destruction of infected cells and releasing of new virus particles into the blood.

**Figure 3:**
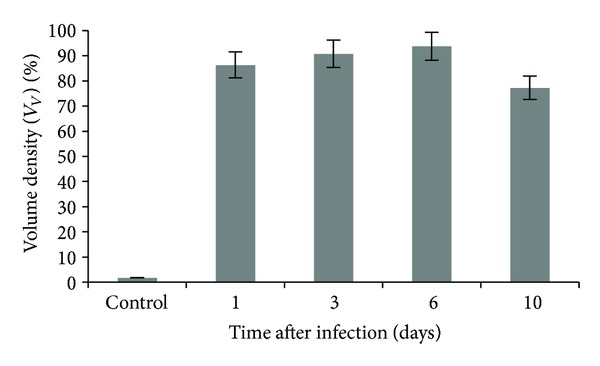
Volume density of the destructive changes in the liver of mice infected with influenza virus A/goose/Krasnoozerskoye/627/05 (A/H5N1).

At the histological examination of the liver of infected animals in the first six days, we registered expansion and congestion of major veins and sinusoids with signs of stasis and hemolysis of erythrocytes. From the first day after infection, we observed hepatocytes in a state of dystrophy, necrosis. Volume density of destructive changes of hepatocytes on day 6 postinfection was the largest and amounted to 93.78% (Figure 3), which indicates the subtotal damage to hepatocytes and the development of liver failure. To day 10 of the experiment, the value of this indicator decreased slightly (Figure 3), which may indicate the activation of repair processes in the surviving animals.

**Figure 4:**
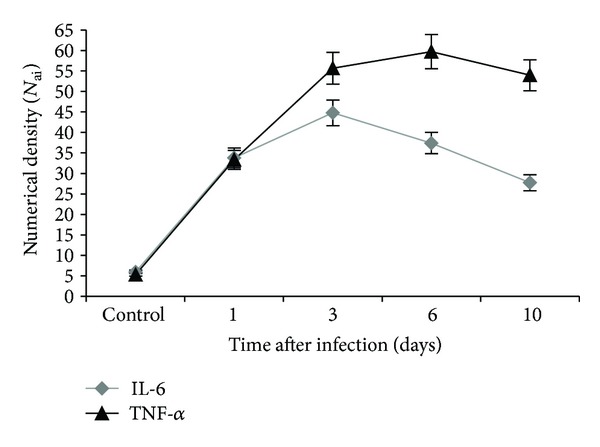
Numerical density of Kupffer cells expressing TNF-*α* and IL-6 in the liver of mice infected with influenza virus A/goose/Krasnoozerskoye/627/05 (A/H5N1).

At the IHC study of cytokine profile, we registered expression of TNF-*α* and IL-6 by Kupffer cells and sinusoidal endothelial cells at the cellular level. The number of liver cells with IL-6 expression was the largest on day 3 of infection and gradually decreased to day 10 of the experiment (Figure 4). The total numeric density of the liver cells with the expression of TNF-*α* was high on the first day and reached its maximum value on day 6. During the whole experiment, Kupffer cells were the dominant type of liver cells expressing proinflammatory cytokines.

To assess the hydrolytic capacity of Kupffer cells of mice infected with A/H5N1 virus, production of lysozyme, cathepsin D, and myeloperoxidase was investigated. Increased expression of these enzymes was observed at all stages of the experiment.

**Figure 5:**
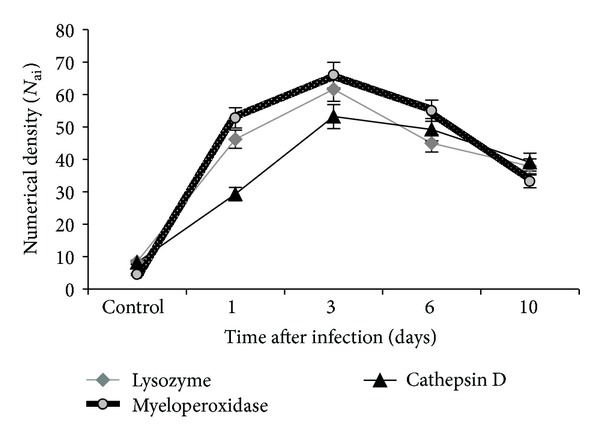
Numerical density of Kupffer cells expressing lysozyme, cathepsin D, and myeloperoxidase in the liver of mice infected with influenza virus A/goose/Krasnoozerskoye/627/05 (A/H5N1).

Already at the first day of infection significant increase of cells expressing lysozyme was recorded in the liver of infected mice, and the vast majority of them were provided by Kupffer cells. The value of this index increased to day 3; then there was an observed reduction of concentration of cells expressing lysozyme till the end of the experiment (Figure 5). Also, lysozyme-secretory activity was detected in endothelial cells, but their numbers were insignificant compared with Kupffer cells. Probably, the prevalence of cells expressing the lysozyme in the early stages after infection has an adaptive nature in connection with a broad spectrum of activity of this enzyme, which belongs to the enzymes of “rapid response” destroying the peptidoglycan envelope viruses and enhancing the effectiveness of phagocytosis [21, 22].

Later, azurophilic granules are mobilized. They contain cathepsins, including cathepsin D as the most common [23]. The number of cells expressing cathepsin D in the liver of mice infected with A/H5N1 virus also reached its maximum value on day 3 of the disease with a gradual decrease to day 10 (Figure 5).

**Figure 6:**
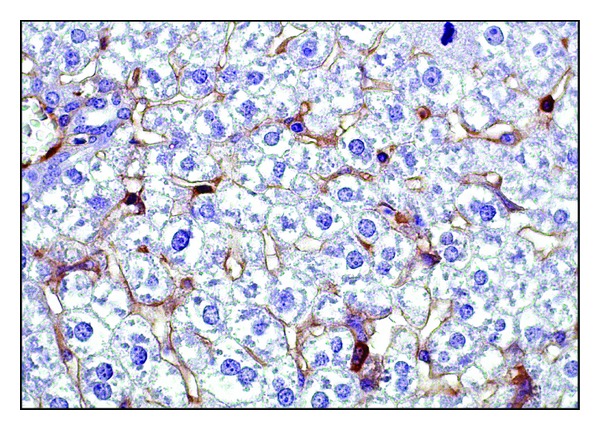
Myeloperoxidase expression by Kupffer cells of the liver of mice infected with A/goose/Krasnoozerskoye/627/05 (A/H5N1) influenza virus. Three days after infection. Immunohistochemical analysis. Magnitude ×630.

The numerical density of cells expressing myeloperoxidase in the liver of infected mice was the highest in comparison with other investigated enzyme levels at all stages of observation, and it also reached a maximum on day 3 of the disease. To day, 10 there was a 1.6-fold decrease of this parameter (Figures 5 and 6). Myeloperoxidase oxidizes cofactors, translating them into an active form, thus providing a powerful microbicidal effect [24]. However, the output of myeloperoxidase in the extracellular space by exocytosis or phagocytic destruction can cause pathological action of the enzyme under the conditions for its development, for example, low pH values during ischemia. The resulting strong oxidants cause tissue damage of the organism [25].

Analysis of the results suggests that in conditions of depletion of lymphoid tissue caused by HPAI H5N1, a special role in protecting the organism from infection belongs to the liver as a key organ of the nonspecific protection.

High HPAI H5N1 virus tropism for vascular endothelial cells of all organs promotes early viremia and generalization of infection. A/H5N1 virus persistence in cells of different histogenesis is associated with a significant activation of cell-mediated immunity, resulting in an increased production of proinflammatory cytokines (TNF-*α* and IL-6) that, on the one hand, is a protective mechanism consisting in the recruitment of immune cells into the focus infection and in the activation of regenerative processes in the organs via growth factors. On the other hand, an excessive accumulation of inflammatory cytokines facilitates the initiation of degradation processes [12].

A/H5N1 virus infection of mice was accompanied by early activation of the expression of myeloperoxidase, lysozyme, and cathepsin D by Kupffer cells. Intracellular proteases are the most important factors of nonspecific protection that provide the intracellular disintegration of the virus proteins. But, apparently, these mechanisms are ineffective for HPAI H5N1 viruses.

High hydrolytic capacity of cells, being an early nonspecific protection factor, at the same time, can be a trigger point to initiate lysis of host cells (secondary alteration) with the development of spread destructive complications in the organs in case of phagolysosome membrane labilization after absorption of viral particles.

Thus, defective antiviral response that triggers a cascade of cytokine-mediated responses, only exacerbates the destructive processes in the liver of experimental animals, which together with insufficient oxygen supply caused by damage of lungs lead to irreversible structural and functional changes and the early development of acute liver failure.
